# Unveiling molecular mechanisms and therapeutic targets in HbH-CS disease: a focus on oxidative stress and mitochondrial dysfunction

**DOI:** 10.1515/biol-2025-1337

**Published:** 2026-06-05

**Authors:** Liuying Nong, Ling Shi, Lihong Pang

**Affiliations:** Department of Prenatal Diagnosis, The First Affiliated Hospital of Guangxi Medical University, Nanning, Guangxi, 530021, China; Department of Reproductive Medicine, Liuzhou Maternity and Child Healthcare Hospital, Liuzhou, Guangxi, 545000, China; Department of Reproductive Medicine, Guangzhou Women and Children’s Medical Center Liuzhou Hospital, Liuzhou, Guangxi, 545000, China

**Keywords:** thalassemia, oxidative damage, mitochondrial function, apoptosis

## Abstract

This study aimed to investigate the molecular mechanisms and therapeutic targets related to oxidative stress and mitochondrial dysfunction in Hemoglobin H-Constant Spring (HbH-CS) disease. HbH-CS differentially expressed genes (DEGs) were selected from a microarray dataset. Oxidative stress-related genes (OSGs) and mitochondrial function-related genes (MiRGs) were retrieved from public databases. Oxidative stress and mitochondrial function-related genes (OMRGs) were defined as the intersection of HbH-CS DEGs, OSGs, and MiRGs. Programmed cell death (PCD) mechanisms and functional enrichment analyses were subsequently investigated. A protein–protein interaction (PPI) network was constructed to identify hub genes, and potential regulatory mechanisms and expression levels of candidate genes were further explored. A total of 98 OMRGs were identified, which were associated with cellular respiration, oxidative stress response, mitochondrial membrane, and apoptotic signaling. Apoptosis was determined to be the primary PCD mechanism. Three hub genes were identified: AKT serine/threonine kinase 1 (AKT1), B cell lymphoma/leukemia-2 (BCL2), and cytochrome *c*, somatic (CYCS). Additionally, RNA-binding motif protein 15B (RBM15B) was recognized as a shared N6-methyladenosine (m^6^A) regulator. The mRNA expression levels of these four genes were significantly downregulated in HbH-CS patients. These findings provide novel insights into the molecular mechanisms and identify potential therapeutic targets for clinical intervention.

## 1 Introduction

Thalassemia is one of the most prevalent and clinically impactful monogenic disorders globally. Hemoglobin H (HbH) disease is caused by functional defects in three of the four α-globin genes, with HbH-CS disease representing one of the most common and severe subtypes in clinical settings. The pathogenesis of HbH-CS disease involves the deletion of two α-globin genes and the CS mutation in the hemoglobin subunit alpha 2 (HBA2) gene, which exhibits higher transcriptional activity than HBA1. Meanwhile, the α^CS^ chain is structurally unstable and easily degraded, thereby leading to an almost complete deficiency of functional α-globin chains in affected individuals.

However, clinical treatments available to date remain limited [1], and both the quality of life and prognosis of these patients call for improvement. The severity of anemia varies significantly among HbH-CS patients, and conventional α-globin genetic defects fail to fully explain this heterogeneity. Accumulation of excess β-globin chains, which possess abnormally high oxygen affinity, exacerbates cellular hypoxia and oxidative stress. Excessive reactive oxygen species (ROS) directly attack mitochondria, triggering mitochondrial dysfunction and further ROS accumulation [2], 3]. When combined with iron overload and cell membrane damage, these factors accelerate erythroid cell death and aggravate hemolysis [4], 5]. However, the regulatory mechanisms underlying oxidative stress and mitochondrial dysfunction in HbH-CS disease remain poorly elucidated. Our study aims to identify the OMRGs and their molecular features in HbH-CS disease, with the findings expected to provide new potential therapeutic targets for this condition.

## 2 Materials and methods

### 2.1 Identification of OMRGs in HbH-CS disease

This study was conducted in accordance with the Declaration of Helsinki (as revised in 2013) and approved by the Medical Ethics Committee of the First Affiliated Hospital of Guangxi Medical University. The HbH-CS group consisted of patients diagnosed with HbH-CS disease who tested negative for β-thalassemia, while the control group comprised individuals with negative results for thalassemia gene testing and no other anemia-causing conditions. Exclusion criteria included severe comorbidities affecting major organ systems, concurrent hematologic disorders (e.g., autoimmune hemolytic anemia, aplastic anemia, leukemia), a history of antipyretic analgesic use, or sulfonamide exposure within the preceding three months, pregnancy or lactation, and any other clinical or laboratory findings deemed incompatible with study participation by the investigators.

In our previous study, erythroid cells were isolated from peripheral blood samples of patients with HbH disease and healthy controls for microarray analysis. Sample preparation, with five biological replicates per group (HbH-CS vs. control), and microarray hybridization were performed following Arraystar’s standard protocols. The HbH-CS DEGs were obtained from the human mRNA and lncRNA epitranscriptomic microarray (8×60K, Arraystar Inc., USA) with thresholds of |log_2_ fold change (logFC)| ≥ 2 and *p* < 0.05. The OSGs were retrieved from the GeneCards database (https://www.genecards.org/) by querying the term “oxidative stress” and applying a relevance score cutoff of >10. The MiRGs were obtained from the Mitoproteome database (http://www.mitoproteome.org/) and the human MitoCarta3.0 database (http://www.broadinstitute.org/mitocarta). The OMRGs were defined as the intersection of the HbH-CS DEGs, OSGs, and MiRGs using the Draw Venn Diagram tool (http://bioinformatics.psb.ugent.be/webtools/Venn/). A Circos heatmap and principal component analysis (PCA) of OMRGs were generated using the OmicStudio tools at https://www.omicstudio.cn/tool.

**Informed consent:** Informed consent was obtained from all individuals included in this study, or their legal guardians or wards.

**Ethical approval:** The research related to human use has been complied with all the relevant national regulations, institutional policies and in accordance with the tenets of the Helsinki Declaration, and has been approved by the Medical Ethics Committee of the First Affiliated Hospital of Guangxi Medical University (No. 2019-KY-GuikeGong-001).

### 2.2 Major PCD mechanisms and functional analysis of OMRGs

Gene sets corresponding to 18 patterns of PCD [6] were intersected with OMRGs to investigate the major PCD mechanisms mediated by OMRGs. Functional analyses of OMRGs in biological process (BP), cellular component (CC), and molecular function (MF) categories were performed using the WebGestalt database (https://www.webgestalt.org). Enriched signaling pathways were further explored from Kyoto Encyclopedia of Genes and Genomes (KEGG), Panther, Reactome, and WikiPathways within this platform. Additionally, the Metascape database (https://metascape.org/gp/index.html#/main/step1) was utilized to analyze Gene Ontology (GO) annotations and signaling pathways associated with OMRGs.

### 2.3 Identification and coordinated functional network of hub genes

A PPI network of OMRGs was constructed using the STRING database (https://cn.string-db.org/) and subsequently sorted by degree value via Cytoscape software [7]. Topological analysis of the PPI network was performed using CytoHubba plugin [8], and hub genes were identified as the intersection of the top 10 genes generated by the MCC, MNC, and Degree algorithms. The GeneMANIA database (http://genemania.org/) was employed to predict genes interacting with hub genes and coordinated biological functions.

### 2.4 Screening for shared m^6^A regulators associated with hub genes

The mRNA expression levels and m^6^A quantity levels of hub genes were visualized using OmicStudio tools at https://www.omicstudio.cn/tool. Through the RNA-binding protein (RBP) binding sites module integrated with individual nucleotide resolution crosslinking and immunoprecipitation (iCLIP) technology in the POSTAR3 database [9], the top five non-redundant RBPs were selected to predict those potentially interacting with each hub gene. Subsequently, a total of 24 m^6^A regulators (comprising 8 writers, two erasers, and 14 readers) were compiled from published literature [10], with details provided in Table A1. By intersecting these 24 regulators with the RBPs of each hub gene and the HbH-CS DEGs, the shared m^6^A regulators associated with hub genes were identified.

### 2.5 Molecular expression characterization of hub genes

The mRNA subcellular localization characteristics of hub genes were analyzed using mRNALocater [11], covering five subcellular components: cell cytoplasm, endoplasmic reticulum, nucleus, mitochondria, and extracellular region. Concurrently, the protein subcellular localizations of these hub genes were analyzed via the CELLO v.2.5 database [12]. All relevant results were visualized via OmicStudio tools.

### 2.6 Validation of clinical samples for target genes

Blood cell isolation was completed within 2 h after blood collection. The separation procedures were performed on a clean bench, with the ambient temperature maintained at 18–22 °C. Mononuclear cells containing nucleated red blood cells (NRBCs) were isolated from peripheral blood using human whole blood mononuclear cell separation medium (Solarbio, China). Following density gradient centrifugation, the blood sample was fractionated into four distinct layers: the top layer contained plasma and platelets, which had the lowest specific gravity; the second layer was a milky-white annular interphase, where NRBCs were concentrated; the third layer consisted of separation medium; and the bottom layer contained erythrocytes and polymorphonuclear granulocytes, with the highest specific gravity. The mononuclear cells in the second layer included CD45-positive white blood cells, predominantly lymphocytes and monocytes, while the expression of transferrin receptor (CD71) was nearly undetectable in mature red blood cells. Based on this characteristic, CD71 MicroBeads and the MiniMACS™ Starting Kit (Miltenyi Biotec, Germany) were employed for the positive selection and enrichment of NRBCs [13], 14].

Total RNA was extracted from samples using TRIzol™ Reagent (Invitrogen, USA), and RNA purity and concentration were determined using a NanoDrop™ One spectrophotometer (Thermo Fisher Scientific, USA). Complementary DNA (cDNA) was synthesized via reverse transcription using HiScript III All-in-one RT SuperMix Perfect for qPCR (Vazyme Biotech, China), with the reaction carried out on a ProFlex™ PCR System (Applied Biosystems, USA). Quantitative real-time polymerase chain reaction (qRT-PCR) was conducted using a StepOnePlus™ Real-Time PCR System (Applied Biosystems, USA), with primer sequences (synthesized by GENEWIZ, China) provided in Table A2. Relative mRNA expression levels of target genes were calculated using the 2^−ΔΔCt^ method.

### 2.7 Statistical analysis

Statistical analyses were performed using GraphPad Prism 9.0 (GraphPad Software, Inc., USA). All experiments were independently repeated at least three times. A *p*-value <0.05 was considered statistically significant.

## 3 Results

### 3.1 Identification of OMRGs in HbH-CS disease

**Figure 1:**
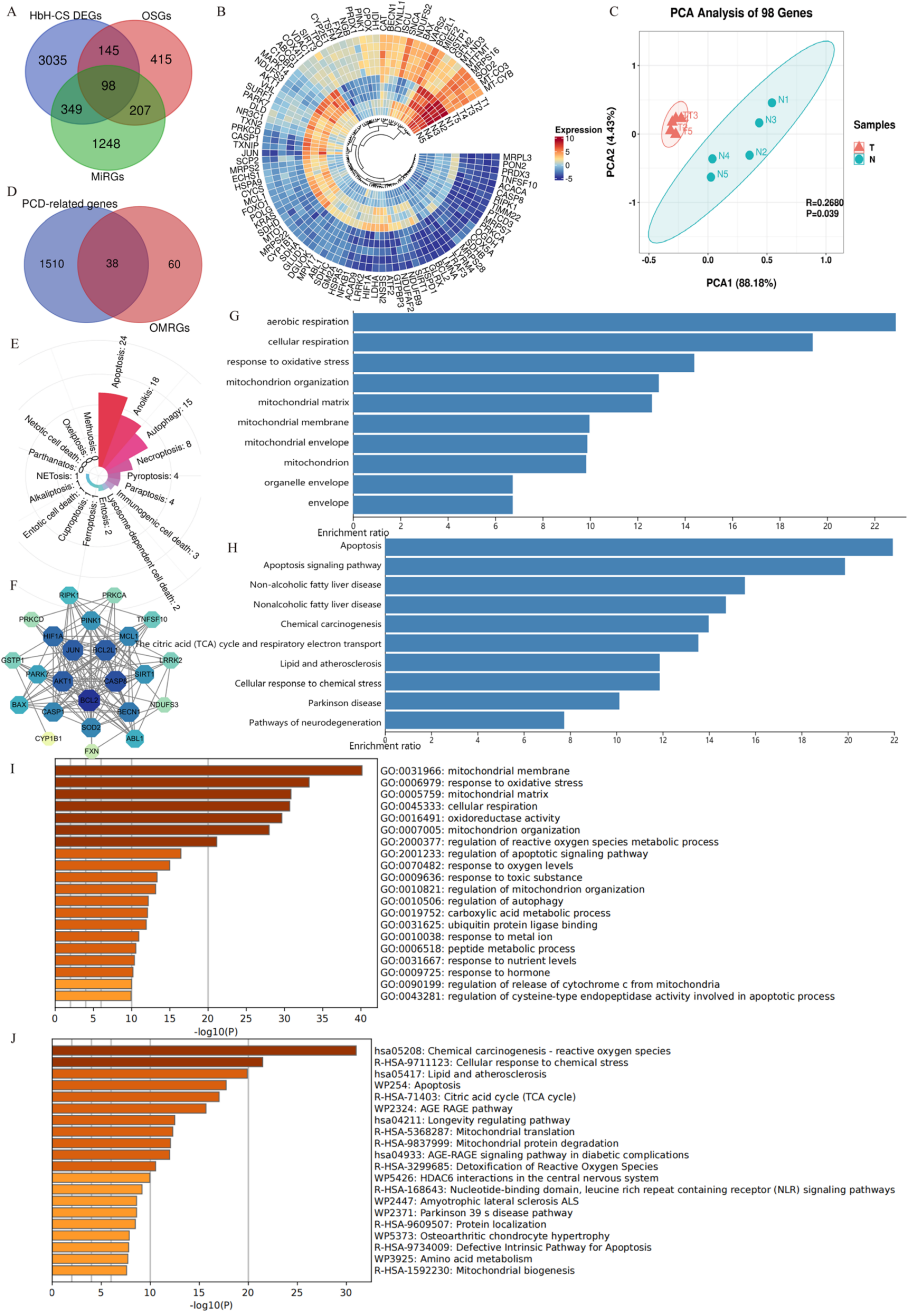
Identification, major PCD mechanisms, and functional analysis of OMRGs. (A) Venn diagram of OMRGs. (B) Circos heatmap of OMRGs. (C) PCA of OMRGs. (D) Venn diagram of PCD-OMRGs. (E) Major PCD mechanisms mediated by OMRGs. (F) PPI network of apoptosis-associated genes. (G) Functional annotations via WebGestalt. (H) Signaling pathways via WebGestalt. (I) GO analysis via Metascape. (J) Signaling analysis via Metascape.

Differential expression analysis of mRNAs between HbH-CS patients and healthy controls identified 3,627 DEGs. A total of 865 OSGs were retrieved from the GeneCards database, while 1,902 MiRGs were acquired by removing duplicates from the Mitoproteome and human MitoCarta 3.0 databases. The intersection of HbH-CS DEGs, OSGs, and MiRGs resulted in the identification of 98 OMRGs in HbH-CS disease, as depicted in the Venn diagram (Figure 1A). As shown in the Circos heatmap, among these OMRGs, 19 were upregulated and 79 were downregulated (Figure 1B). PCA of the OMRGs revealed that samples from the HbH-CS group (Group T) and the control group (Group N) tended to cluster within their respective groups, indicating good intra-group reproducibility. Furthermore, the significant separation between the two groups highlighted the strong representativeness of these OMRGs in HbH-CS (Figure 1C).

### 3.2 Major PCD mechanisms and functional analysis of OMRGs

A total of 1548 PCD-related genes representing 18 cell death mechanisms were intersected with 98 OMRGs, leading to the identification of 38 PCD-OMRGs (Figure 1D). Further analysis revealed that 24 apoptosis-associated genes accounted for the largest proportion among these PCD-OMRGs (Figure 1E). The PPI network of these 24 genes was sorted by degree value, with darker node colors indicating higher importance (Figure 1F). GO annotation results from WebGestalt were mainly associated with aerobic respiration, cellular respiration, oxidative stress response, and mitochondrial structure (Figure 1G). Concurrently, the signaling pathways implicated were principally linked to apoptosis, non-alcoholic fatty liver disease (NAFLD), chemical carcinogenesis, tricarboxylic acid (TCA) cycle, and respiratory electron transport (Figure 1H). Significant GO terms from Metascape included mitochondrial membrane, response to oxidative stress, mitochondrial matrix, cellular respiration, and oxidoreductase activity (Figure 1I). Concurrently, the signaling pathways identified were related to chemical carcinogenesis-reactive oxygen species, cellular responses to chemical stress, lipid metabolism and atherosclerosis, apoptosis, and TCA cycle (Figure 1J).

### 3.3 Identification and coordinated functional network of hub genes

**Figure 2:**
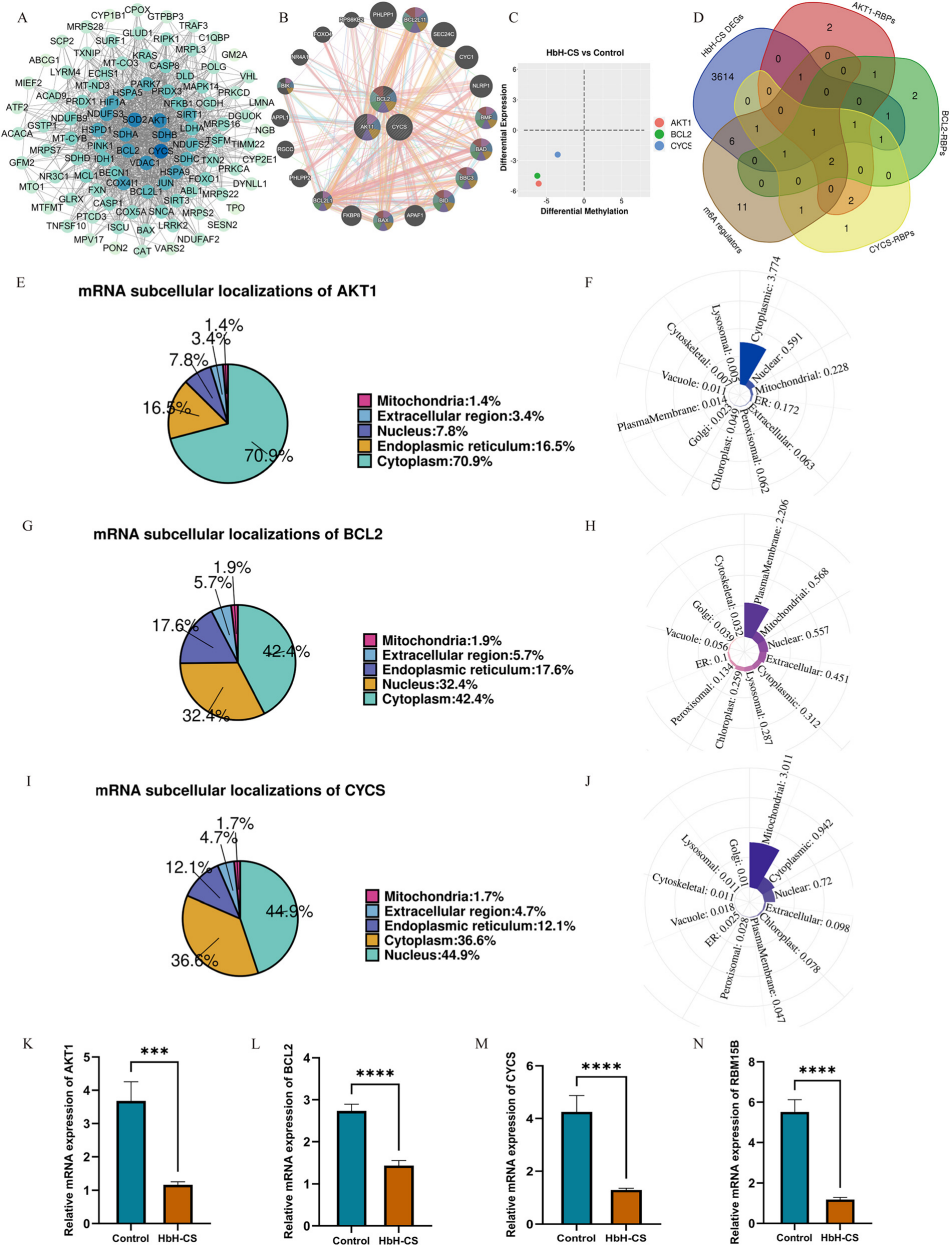
Identification, expression characterization, and validation of target genes. (A) OMRGs-PPI network by cytoscape. (B) Coordinated functional network of hub genes in OMRGs. (C) A joint analysis of mRNA expression levels and m^6^A quantity levels of hub genes. (D) Screening of candidate shared m^6^A regulators among hub genes. (E–J) mRNA and protein subcellular localizations of AKT1, BCL2, and CYCS. (K–N) Clinical expression levels of AKT1, BCL2, CYCS, and RBM15B verified by qRT-PCR. ****p < 0.0001.

The OMRGs-PPI network was constructed using the STRING database and visualized using Cytoscape (Figure 2A). The top 10 candidate genes were identified using the MCC, MNC, and Degree algorithms integrated in CytoHubba plugin, with details provided in Table A3. Subsequent intersection analysis of these candidate gene sets yielded three hub genes: AKT1, BCL2, and CYCS. A coordinated functional network encompassing 23 genes (including the three hub genes) was constructed via GeneMANIA, which was primarily associated with the release of cytochrome *c* from mitochondria, apoptotic mitochondrial changes, regulation of mitochondrion organization, regulation of intrinsic apoptotic signaling pathway, and mitochondrial outer membrane permeabilization (Figure 2B).

### 3.4 Screening for shared m^6^A regulators associated with hub genes

A joint analysis of mRNA expression levels and m^6^A quantity levels of the three hub genes, based on our transcriptional microarray data, was presented in Figure 2C. The m^6^A quantity levels of CYCS, AKT1, and BCL2 were all decreased, details were provided in Table A4. An intersection analysis was performed among HbH-CS DEGs, m^6^A regulators, and RBPs associated with the three hub genes, implicating RBM15B as a candidate shared m^6^A regulator (Figure 2D). Correspondingly, the mRNA expression levels of AKT1, BCL2, CYCS, and RBM15B were downregulated in HbH-CS patients (Table A5).

### 3.5 Molecular expression characterization of hub genes

AKT1 mRNA was predominantly localized in the cytoplasm (Figure 2E), which was consistent with the cytoplasmic localization of AKT1 protein (Figure 2F). BCL2 mRNA was primarily distributed in the cytoplasm and nucleus (Figure 2G), whereas BCL2 protein was concentrated at the plasma membrane (Figure 2H). CYCS mRNA was mostly located in the nucleus and cytoplasm (Figure 2I), with CYCS protein predominantly enriched in the mitochondria (Figure 2J).

### 3.6 Validation of clinical samples for target genes

A total of 16 HbH-CS patients and 16 healthy controls were enrolled for the collection of peripheral blood samples. qRT-PCR analysis revealed that the mRNA expression levels of AKT1, BCL2, CYCS, and RBM15B were significantly downregulated in HbH-CS patients compared with the control group (Figure 2K–N).

## 4 Discussion

In this study, transcriptomic microarray profiling was conducted to characterize the mRNA expression signature of HbH-CS disease, leading to the identification of 98 representative OMRGs. Blood transcriptome-based molecular diagnostic strategies have been widely adopted to identify disease-specific biomarkers and stress-induced biological phenotypes, owing to their clinical application merits, including convenient sample acquisition and timely monitoring [15], 16]. Subsequently, functional annotation and enrichment analyses were performed, which revealed that these OMRGs were predominantly linked to cellular respiration, oxidative stress response, mitochondrial membrane, apoptotic signaling, and the TCA cycle.

A key pathological feature of HbH-CS disease is the accumulation of free β-globin chains in erythroid cells, which facilitates the formation of non-functional β4 tetramers (HbH) with abnormally high oxygen affinity. Notably, this aberrant β-chain assembly exhibits poor oxygen delivery capacity [17], thereby exacerbating intracellular hypoxic stress. Overproduction of ROS induces oxidative damage to mitochondria and other essential cellular organelles. Furthermore, the deposition of HbH inclusion bodies on the erythroid cell membrane impairs membrane structural stability. Persistent chronic hemolysis – a cardinal feature of HbH-CS, further promotes systemic iron accumulation and subsequent iron overload in affected patients. As a critical catalyst in free radical generation and Fenton reactions, iron exacerbates oxidative damage and ferroptosis [18], ultimately compromising erythroid cell function and survival.

Further analysis revealed that apoptosis is likely the predominant mechanism underlying PCD-OMRGs. As a genetically regulated form of PCD, apoptosis can be triggered by any stimulus that induces oxidative stress and mitochondrial dysfunction. Although patients with HbH-CS exhibit significant hemolysis, extensive erythroid cell death does not elicit a robust inflammatory or prominent allergic response *in vivo*. This phenomenon may be partially attributed to non-inflammatory apoptosis, which helps maintain relative internal environmental homeostasis – consistent with the clinical manifestations observed in affected patients. Intracellular mitochondria are critical for erythroid cells, remaining functionally active from nucleated erythrocytes to orthochromatic erythroblasts to support hemoglobin synthesis. Furthermore, mitochondrial dysfunction exerts detrimental effects on erythroid cell maturation [19]. Excessive ROS disrupt redox homeostasis, impair mitochondrial function, and accumulate to ultimately induce erythroid cell damage, enhance hemolysis, and exacerbate progressive anemia [20].

Three hub genes – namely AKT1, BCL2, and CYCS – were identified from the OMRGs. An interaction network comprising these hub genes and 20 additional associated genes was constructed, revealing that the predominant biological functions are linked to the release of cytochrome *c* from mitochondria, apoptotic mitochondrial changes, regulation of mitochondrion organization, and regulation of intrinsic apoptotic signaling pathway. To investigate the potential roles of these hub genes in HbH-CS disease, their mRNA expression profiles and protein subcellular localizations were analyzed. Both AKT1 mRNA and protein are predominantly localized to the cytoplasm. AKT1 serves as a central node in the PI3K/AKT signaling pathway and has been implicated as a key mediator of cell survival [21]. Activation of the AKT1-dependent signaling axis can protect cells from oxidative stress [22]. The BCL2 protein is predominantly localized to the outer mitochondrial membrane. B-cell lymphoma-2 (Bcl-2) acts as an oxidative stress-responsive protein [23] and a key regulator of apoptosis. Moreover, Bcl-2 exerts its anti-apoptotic function by preserving mitochondrial integrity [24], and upregulated Bcl-2 expression has been shown to suppress the mitochondrial apoptotic pathway [25]. CYCS, which encodes cytochrome *c* (Cyt *c*) – a hemoprotein essential for mitochondrial electron transport and apoptosis, possesses certain antioxidant and antiapoptotic capacities [26]. It can scavenge excessive intracellular ROS and preserve physiological ROS homeostasis [27], thereby alleviating oxidative damage. Furthermore, cytochrome *c* deficiency disrupts respiratory chain function and oxidative phosphorylation, reducing ATP synthesis and aggravating superoxide anion overproduction, which ultimately triggers apoptosis [28].

Furthermore, integrated multi-omics analyses demonstrated a concurrent decrease in both m^6^A modification levels and mRNA expression of the three hub genes. Importantly, this study proposes that RBM15B, which encoded by the OTT3 gene on chromosome 3 and classified as an RBP involved in epitranscriptomic modification, may act as a shared m^6^A regulator for these hub genes. RBM15, encoded by the OTT1 gene on chromosome 1, is recognized as the ortholog of RBM15B. High similarity has been reported between RBM15 and RBM15B in terms of coding sequence (CDS) and protein domain architecture [29]. Notably, RBM15 is recognized as an essential regulator of normal hematopoietic stem cell (HSC) development [30]. At present, however, the role of RBM15B in hematopoiesis and HbH-CS disease remains poorly explored. Our findings suggest that RBM15B modulates the mRNA expression of these hub genes, potentially through its characterized role in m^6^A-dependent post-transcriptional regulation. Clinical validation showed that the mRNA expression levels of AKT1, BCL2, CYCS, and RBM15B were reduced in HbH-CS patients relative to the controls, consistent with the transcriptomic microarray expression trend observed in this study.

Beyond genetic determinants, other exacerbating factors in HbH-CS pathogenesis warrant in-depth investigation. The extent of oxidative damage to erythrocytes has been established to influence thalassemia progression [31]; furthermore, maintaining erythrocyte redox homeostasis has been shown to prevent the exacerbation of hemolytic anemia [32]. It has been demonstrated that reducing ROS and improving mitochondrial function can alleviate apoptosis and hemolysis in both murine models and erythrocyte precursors derived from β-thalassemia patients [33], 34]. Additionally, plasma proteins associated with hemolysis, oxidative stress, and hemoglobin degradation are differentially expressed in HbH-CS patients compared with individuals with other HbH disease subtypes and controls [35]. Erythroid cells sustain heightened oxidative damage in HbH-CS disease, leading to functional impairment and shortened cellular lifespan – a phenomenon that exhibits age-related exacerbation. Consequently, mitigating oxidative damage and restoring mitochondrial function might represent a therapeutic approach for HbH-CS disease.

We provided a potential molecular landscape of oxidative stress and mitochondrial dysfunction in HbH-CS disease. Notably, we identified RBM15B as a potential shared m^6^A regulator of AKT1, BCL2, and CYCS in HbH-CS disease – an observation that has not been previously reported. This finding advances our understanding of epitranscriptomic regulation in thalassemia. Despite these novel insights, the present study has several limitations. First, the sample size was relatively small, which may restrict the generalizability of our results. Second, the study relied primarily on transcriptomic data, which may not fully capture the functional status of these molecules. Further investigations are warranted to validate these targets in multi-center cohorts, utilize *in vitro* and *in vivo* models for functional validation, and confirm their clinical utility in the management of HbH-CS disease. In particular, future research should focus on elucidating the specific molecular mechanisms by which RBM15B regulates these hub genes through m^6^A modification.

Our findings identify AKT1, BCL2, CYCS, and RBM15B as potential biomarkers and therapeutic candidates, which may be involved in regulating mitochondrial function and oxidative damage, thereby providing a potential therapeutic strategy for HbH-CS disease. Leveraging these biomarkers to identify high-risk patients and initiating targeted therapy at an early stage may delay erythrocyte destruction, reduce complications related to chronic hemolysis, diminish transfusion needs, and ultimately enhance long-term quality of life.

## 5 Conclusions

In conclusion, three hub genes (AKT1, BCL2, and CYCS) were identified as potential mediators regulating oxidative stress and mitochondrial function. Furthermore, RBM15B was recognized as a key m^6^A regulatory factor associated with the progression of HbH-CS disease. The present study enriches the current understanding of the transcriptomic characteristics in HbH-CS disease. Further in-depth studies are required to elucidate the underlying molecular mechanisms and validate effective therapeutic targets.

## Appendix A

**Table A1:** The m^6^A regulators.

Classification	m^6^A regulators
Writers	CBLL1, METTL3, METTL14, RBM15, RBM15B, VIRMA, WTAP, ZC3H13
Erasers	ALKBH5, FTO
Readers	ELAVL1, FMR1, HNRNPA2B1, HNRNPC, IGF2BP1, IGF2BP2, IGF2BP3, LRPPRC, RBMX, YTHDC1, YTHDC2, YTHDF1, YTHDF2, YTHDF3

**Table A2:** Primer sequences for RT-qPCR.

Gene	Forward primer (5′-3′)	Reverse primer (5′-3′)
β-actin	GCA​CTC​TTC​CAG​CCT​TCC​TT	CGT​ACA​GGT​CTT​TGC​GGA​TG
AKT1	CAC​CTT​CAT​CAT​CCG​CTG​CCT​G	TCT​CCT​CCT​CCT​CCT​GCT​TCT​TG
BCL2	CCT​GTG​GAT​GAC​TGA​GTA​CC	CTT​CAG​AGA​CAG​CCA​GGA​GA
CYCS	ACT​CTT​ACA​CAG​CCG​CCA​AT	GTC​TGC​CCT​TTC​TTC​CTT​CTT​C
RBM15B	ATG​AGG​AAC​GGA​GTA​GGA​CCA​AG	GTC​TGC​TGT​TGC​TGA​GGG​AGT​T

**Table A3:** Top 10 genes of OMRGs identified by CytoHubba.

Rank	Algorithm		
	MCC	MNC	Degree
1	CYCS	CYCS	CYCS
2	AKT1	SOD2	SOD2
3	BCL2	AKT1	AKT1
4	BCL2L1	SDHB	SDHB
5	SIRT1	VDAC1	VDAC1
6	JUN	BCL2	BCL2
7	HSPA5	SDHA	SDHA
8	CASP8	NDUFS3	COX4I1
9	MCL1	COX4I1	NDUFS3
10	CASP1	HSPA9	HSPA9

**Table A4:** m^6^A quantity levels of hub genes from microarray data.

Gene symbol	Regulation	Log_2_FC	P-value	FDR
AKT1	Hypo	−6.044034569	0.000047101	0.002343154
BCL2	Hypo	−6.22197778	0.000062111	0.00256982
CYCS	Hypo	−3.511041803	0.004379448	0.016183269

**Table A5:** The mRNA expression levels of target genes from microarray data.

Gene symbol	Regulation	Log_2_FC	P-value	FDR
AKT1	Down	−5.279785927	0.000432736	0.012203592
BCL2	Down	−4.50504045	0.000665818	0.014223047
CYCS	Down	−2.413772555	0.024638925	0.075611014
RBM15B	Down	−4.465186709	0.001250572	0.01841846
